# The Host Microenvironment Influences Prostate Cancer Invasion, Systemic Spread, Bone Colonization, and Osteoblastic Metastasis

**DOI:** 10.3389/fonc.2014.00364

**Published:** 2014-12-15

**Authors:** Sourik S. Ganguly, Xiaohong Li, Cindy K. Miranti

**Affiliations:** ^1^Program for Skeletal Disease and Tumor Metastasis, Laboratory of Tumor Microenvironment and Metastasis, Center for Cancer and Cell Biology, Van Andel Research Institute, Grand Rapids, MI, USA; ^2^Program for Skeletal Disease and Tumor Metastasis, Laboratory of Integrin Signaling and Tumorigenesis, Center for Cancer and Cell Biology, Van Andel Research Institute, Grand Rapids, MI, USA

**Keywords:** prostate, cancer, tumor microenvironment, bone metastasis, EMT

## Abstract

Prostate cancer (PCa) is the second leading cause of cancer death in men worldwide. Most PCa deaths are due to osteoblastic bone metastases. What triggers PCa metastasis to the bone and what causes osteoblastic lesions remain unanswered. A major contributor to PCa metastasis is the host microenvironment. Here, we address how the primary tumor microenvironment influences PCa metastasis via integrins, extracellular proteases, and transient epithelia-mesenchymal transition (EMT) to promote PCa progression, invasion, and metastasis. We discuss how the bone-microenvironment influences metastasis; where chemotactic cytokines favor bone homing, adhesion molecules promote colonization, and bone-derived signals induce osteoblastic lesions. Animal models that fully recapitulate human PCa progression from primary tumor to bone metastasis are needed to understand the PCa pathophysiology that leads to bone metastasis. Better delineation of the specific processes involved in PCa bone metastasize is needed to prevent or treat metastatic PCa. Therapeutic regimens that focus on the tumor microenvironment could add to the PCa pharmacopeia.

## Foreward

Prostate cancer (PCa), one of the most common non-skin cancers, results in the death of over a quarter million men annually world-wide (1) and 2.7% American men are estimated to die from this disease in their lifetime (2). The majority of PCa deaths are due to the development of metastatic disease, 80% of which is primarily localized in the bone (3). Furthermore, PCa induces an osteoblastic reaction within the bone, which is rarely observed with other bone-metastatic cancers.

For the patient who presents with metastatic disease, as evidenced by bone lesions detected by X-ray or it is suspected based on a high Gleason score, the first line of treatment after surgical removal of the primary tumor is androgen-deprivation therapy (ADT). The majority of prostate tumors require androgen for their growth and survival (4). Thus, the initial metastatic tumor burden in a patient can be essentially eliminated and they appear to enter remission. But the unfortunate fact is that some subpopulation of these cells either harbor or develop resistance to ADT and the tumor rapidly regrows.

Despite lack of evident dependence on circulating androgen, these castration-resistant tumors are still highly addicted to androgen and/or its cognate receptor, AR (5–8). Evidence for this is provided by several observations. First, second generation enhanced anti-androgen therapies, such as Enzalutamide, are effective, even if only for a while, in patients who failed the first round of ADT (5). Second, recent evidence indicates the tumor itself turns on androgen synthesis, so it no longer needs circulating androgen (9, 10). The successful use of drugs, such as Abiraterone (11), that target enzymes in the androgen synthetic pathway are also effective, again albeit for a short time, in patients who failed ADT. Third, the retention, mutation, and amplification of AR that accompanies ADT-resistant tumors indicate a heavy dependence on AR for the survival and continued persistence of these tumors. Several mutations in AR are known to confer enhanced function and include binding to other steroids or deletion of the ligand binding domain resulting in constitutive activation (12, 13). Fourth, AR could independently enhance invasion and metastasis through non-classical steroid receptor signaling mechanisms (14, 15). Currently, there are no approved therapies available that address these latter two events.

Development of additional therapeutic regimes to target metastatic tumors remains severely limited by the lack of knowledge about (1) what triggers PCa metastasis in the first place, (2) why it displays such a predilection for the bone, and (3) why it induces an osteoblastic bone phenotype. The molecular events thought to be involved in these three processes share a common theme; i.e., interactions with the host, often referred to as the tumor microenvironment. Current approved therapies are highly focused on targeting events occurring intrinsically in the tumor and do not fully consider the contributions of the host. Thus, better understanding of the host and tumor interactions that trigger and drive metastatic processes could provide additional avenues for therapeutic intervention. In this review, we will discuss various possible strategies by which PCa cell interactions with the surrounding tumor microenvironment influence the development of metastases, homing toward the bone, and the development of osteoblastic lesions.

## Tumor Microenvironment in Promoting Prostate Cancer Metastasis

### Introduction

It is widely accepted that the tumor microenvironment, or stromal compartment, is biologically heterogeneous, consisting of various cell types, such as fibroblasts, endothelial cells, and immune cells, along with growth factors and cytokines, and numerous extracellular matrix (ECM) components. Paracrine signals from these factors released by the tumor activate signaling and gene expression in the neighboring cells and vice versa, ultimately setting up a cycle of reinforcement and continued signal propagation. Interactions between the cancer cells and this stromal compartment are required for invasion, angiogenesis, and metastasis of cancer cells to ectopic sites (16–18). The factors thought to drive the metastatic progression of PCa and to play an important role in the interaction of the tumor with its microenvironment are discussed below.

Olumi et al. (18), was the first to demonstrate the dependency of PCa development on the underlying fibroblasts. It was recognized that fibroblasts found near tumors, i.e., carcinoma-associated fibroblasts (CAFs), were fundamentally different from those in non-tumorigenic samples (19, 20). When CAFs isolated from human PCa patients were mixed with initiated human non-tumorigenic prostate epithelial cells, this was sufficient to initiate tumorigenesis. Normal fibroblasts lacked this capacity, implicating the importance of tumor-induced fibroblast effects feeding back on the initiated tumor cells. In another study, prostate stromal cells could replace Matrigel in LNCaP subcutaneous xenografts to promote tumor growth. One effect of the stromal cells was to promote angiogenesis (21). The stromal compartment of the normal prostate gland is full of smooth muscle cells. However, in PCa lesions, there was a dramatic loss of smooth muscle cells that were replaced by cells displaying myofibroblast characteristics, i.e., expression of Vimentin and increased production of matrix remodeling enzymes like Collagen I and Tenascin (22). This remodeling of the ECM and invasion of tumor cells into the surrounding stromal compartment define a cancerous lesion, as opposed to benign disease. The interaction of the prostate tumor cells with the remodeled matrix and the contribution of the tumor cells themselves to this process are critical first steps in the movement of tumor cells out of their normal niche.

### Integrins in PCa progression

Integrins are a large family of cell-surface glycoproteins, which form heterodimeric adhesion receptors. Integrins bind to a number of ECM components and regulate cytoskeletal organization to maintain cell shape and facilitate migration. These interactions also regulate cell survival, proliferation, adhesion, migration, and invasion (23, 24). PCa initiation and progression is accompanied by preferential expression of integrin α6β1, reduction in integrin α3β1, and complete loss of integrin β4 (25). Integrin α6, a laminin receptor is associated with poor patient prognosis and increased metastasis in a wide range of cancers (26, 27). In the normal prostate, integrin α6 complexed with integrin β4 is present at the basal cell/stromal interface; however, loss of integrin polarity occurs during progression of PIN to invasive cancer where basal cells are lost and integrin α6 complexed with integrin β1 abnormally appears in the luminal-like compartment (28).

Integrin α6β1 was shown to play two major roles in PCa, promoting cell survival and facilitating invasion and metastasis (28–30). Within the normal prostate epithelium, integrin expression is limited to the basal cells, being absent from the AR-expressing luminal cells. However, during PCa development, integrin α6β1 becomes co-expressed with AR in the tumor cells (31). AR directly binds the integrin α6 promoter and induces the expression of integrin α6β1, while simultaneously decreasing integrin β4 expression (29). Adhesion of PCa tumor cells to laminin to engage α6β1 promoted AR-dependent survival of the cells. Survival was mediated through AR-induced integrin α6β1 and subsequent activation of NF-κB and Bcl-xL expression (29). This AR/α6β1 pathway was active in metastatic cell lines, and elevated in castration-resistant cells. Laminins are abundant in the bone microenvironment, with Laminin-10 being the most highly expressed (32, 33), thus providing a mechanism for activating the integrin α6β1 survival pathway in the bone. Elevated NF-κB activity plays a critical role in PCa progression (34, 35). Another study demonstrated that secretory proteins from prostate neuroendocrine cells activate NF-κB signaling in the tumor cells, which in turn transcriptionally activates AR in the tumor cells to promote castration-resistant cell growth (36). The potential role of integrin α6β1 in this process remains to be determined. It was previously shown that the integrin β1a variant is expressed in PCa tumors, while the β1c variant is present in normal tissue (37). Signaling through the β1c variant was shown to suppress p27kip, a major negative cell cycle regulator and tumor suppressor known to be disregulated in PCa. Thus, a potential α6β1a variant may contribute to PCa proliferation.

Integrin α6 remains the primary integrin expressed in lymph node and soft tissue metastases, indicating high retention and selection for this integrin during metastasis (38). Cleavage of integrin α6 by uPA is associated with invasive PCa, the cleavage product is detected in tumors but not in normal prostate tissue, and promotes PCa cell invasion and migration on laminin (39, 40). Furthermore, inhibiting integrin α6-mediated adhesion or cleavage delayed experimental lung metastasis (28, 41), reduced bone growth in mouse femurs, and increased responses of metastatic PCa cells to ionizing radiation (42, 43).

Laminin integrins like α6β1 and α3β1 associate with transmembrane scaffold and membrane organizing molecules called tetraspanins (44). Tetraspanin KAI/CD82 was first identified as a metastasis suppressor in PCa (45). Loss of CD82 in human PCa correlates with poor prognosis, but by itself is not sufficient to predict metastasis (46). Studies in PCa cell lines *in vitro* demonstrate that CD82 is capable of suppressing integrin-based functions including signaling, migration, and invasion (47–50). CD82 regulates the internalization and turnover of integrins and suppress integrin signaling through Met and Src (47, 48). Its role in suppressing integrin functions was further validated in KO mice. However, the dependency on integrins for its metastasis suppressive functions did not prove to be valid (CKM, unpublished data). Furthermore, while restoration of CD82 to metastatic cells suppresses metastasis, it loss in primary PCa is not sufficient to induce metastasis in genetically engineered mice (CKM, unpublished data). Thus, additional factors or as yet unidentified mechanism is involved in CD82 suppression of metastasis. A more likely target is its role in promoting cell–cell adhesion (51), discussed further in the EMT section.

The importance of integrin α2β1 in PCa metastasis is also emerging (31). Integrin α2β1 binds collagen, another major component of the prostate basement membrane and bone microenvironment. Manipulation of integrin α2 expression in LNCaP cells demonstrated a direct correlation between integrin α2 expression and the ability to grow in the bone (52). RANKL expression in PCa cells enhances bone metastasis. Integrin α2 integrin expression and function was stimulated in PCa cells overexpressing RANKL (53).

Thus, many studies support the importance of integrins in PCa development and progression by promoting, survival, proliferation, invasion, and metastasis. Targeting specific integrins and their matrix interactions may provide a way to prevent metastatic bone PCa.

### Proteases in PCa tumor invasion and metastasis

**Figure 1:**
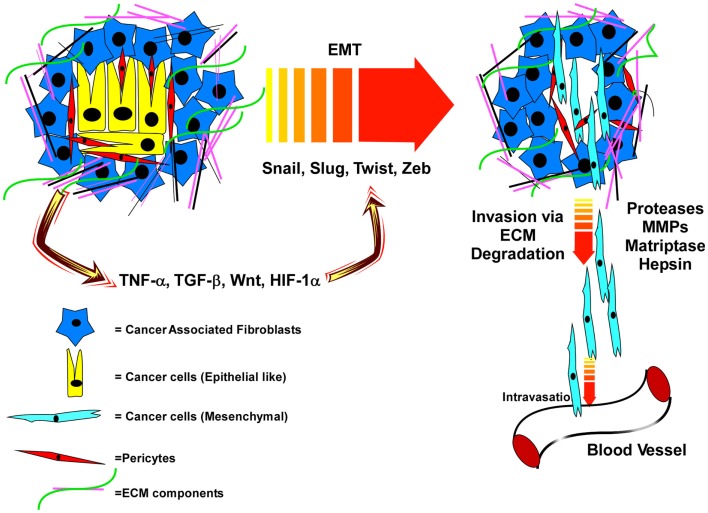
**Interactions of PCa cells with an extracellular matrix that is remodeled by cancer-associated fibroblasts, and soluble factors and proteases released within the tumor microenvironment induce EMT and subsequent invasion and dissemination of cancer cells**. In the primary tumor microenvironment, the epithelial cancer cells are surrounded by the cancer-associated fibroblasts (CAF), pericytes, and various extracellular matrix (ECM) proteins. This tumor microenvironment produces various factors like TNFα, TGFβ, Wnt, and HIF-1α which promote EMT via up-regulation of specific transcription factors. EMT programing leads to a mesenchymal phenotype of the cancer cells and with the help of various proteases (MMPs, Matriptase, Hepsin), the cancer cells cleave the ECM, break away from the tumor microenvironment and intravasate into the blood vesicles to travel to distal organs.

Breakdown of the basement membrane surrounding the prostatic ducts and invasion of prostate cells into the stromal compartment defines the pathology of prostate adenocarcinoma. Proteases that mediate basement membrane and stromal ECM degradation are crucial for this process and represent the first steps toward metastatic dissemination (Figure 1). Penetration into and out of the vasculature and lymphatics similarly requires proteases (54, 55).

Type II transmembrane serine proteases, like Matriptase and Hepsin, which are important for normal epithelial tissue development and repair, contribute to the breakdown of the basement membrane in PCa (56). Hepsin is dramatically up-regulated in PCa and represents one of the most highly overexpressed proteins in PCa microarrays (57). Elevated Hepsin expression is associated with high Gleason score and poor clinical outcome (55, 58, 59). Hepsin overexpression in the prostate epithelium causes disorganization of the basement membrane and its overexpression in PCa cell lines promotes lymph node metastasis (57). Hepsin reportedly cleaves and activates pro-HGF produced by activated fibroblasts within the stroma, thereby activating the receptor tyrosine kinase Met signaling pathway; a known activator of epithelial cell scattering, migration, and invasion (60). However, it was also reported that another Hepsin target is Laminin-5, a major component of the prostatic basement membrane, which is lost during PCa development (61).

Matriptase is expressed in many cancers and increased expression is seen in primary prostate tumors and metastatic lesions (62–64). One of its reported targets is Laminin-5, deposited in the prostatic duct basement membrane primarily by the basal epithelial cells. Laminin-5 expression is lost in PCa, coinciding with the loss of its primary receptor, integrin β4 (65). Active degradation combined with loss of secretion within the emerging PCa cells likely accounts for the lack of Laminin-5 in PCa tumors. Whether Laminin-10, another component of the prostate basement membrane, which is not lost during PCa development, is also cleaved by Matriptase or Hepsin is not known.

While Hepsin expression is apparently not controlled by AR, both Matriptase expression and its cleavage is highly controlled by androgen signaling (66). Rapid cleavage within minutes of androgen stimulation, mediated by Src signaling, is followed by a more long term increase in new Matriptase mRNA, thus providing a mechanism for replenishing depleted pools. Because Matriptase is essential for detachment of epithelial suprabasal cells during skin differentiation (67), and the AR-positive luminal cells also must detach upon differentiation, it is highly likely that Matriptase is also important in prostate epithelial differentiation. That AR, within the newly emerging luminal cells, can control integrin expression and a protease that degrades basement membrane has interesting implications about how AR contributes to preserving a luminal phenotype and may promote loss of basal cells during PCa development (68).

Two other proteases, which are known direct AR transcriptional targets, PSA and TMPRSS2, are normally secreted into the lumens of prostatic ducts (69, 70). However, due to loss of epithelial polarization and invasion into stromal areas, these enzymes are now also present within the tumor microenvironment (71). Their relative importance in PCa development or progression has remained largely undetermined.

The most extensively studied proteases linked with invasion and metastasis are the matrix-metalloproteinases (MMP). MMPs are involved in the degradation of the stromal ECM components such as Collagen and Fibronectin (72, 73). In normal tissues, MMPs play a major role in ECM remodeling involved in development and tissue repair. Their misregulation contributes to many disease states, including rheumatoid arthritis, pulmonary emphysema, and tumor invasion and metastasis (74–77). Active MMPs are secreted mainly from the cells in the tumor microenvironment, such as connective tissue, fibroblasts, endothelial cells, osteoblasts, macrophages, and neutrophils but also by cancer cells. Active MMPs are used by cancer cells to invade the stromal compartment both at the primary site and at metastatic sites (75, 76).

In PCa, MMP-2 and -9 are considered useful prognostic markers and these MMPs promote invasion and metastasis of PCa cells (78, 79). Elevated levels of MMP-2/9 in serum or plasma are correlated with high Gleason score (78–80). Overexpression of MMP-1 promoted PCa cell invasion and experimental metastasis, and inhibition of MMP-1 activity decreased PCa tumor growth in mice, indicating the importance of MMP-1 in regulating PCa invasion and metastasis (81). Induction of PCa invasion by MMP-9 is mediated through cleavage and subsequent inactivation of the serpin protease nexin-1 (PN-1). PN-1 is known to inhibit urokinase plasminogen activator (uPA) and thus inhibits PCa progression and metastasis (82). uPA and its receptor (uPAR) promote PCa metastasis, as down regulation of uPA or uPAR inhibited PCa cell invasion and metastasis (83, 84). When this is coupled with the reported role of uPA in cleaving integrin α6 (39), it becomes apparent how concerted efforts of proteases and integrin-based cell adhesion work together to promote invasion and metastasis.

Attempts to therapeutically target the MMPs, as a whole class, failed in clinical trials; resulting in worse outcomes (85). The lack of specificity to specific MMPs and the unforeseen protective role of some MMPs are thought to have contributed to the failure. Thus, there has been much resistance to trying to identify specific MMP inhibitors. On the other hand, preclinical testing of a small molecule Hepsin inhibitor demonstrated it blocked PCa metastasis in a genetic mouse model (57). Curcumin has the capacity to inhibit androgen induced Matriptase activation and displays anti-metastatic properties (86). Antibodies that block Matriptase cleavage have been reported (87), and a natural protein product produced by bacteria, Ecotin, is a natural Matriptase inhibitor (88), either of which may offer a therapeutic advantage. More work in defining the protease targets and mechanisms for inducing invasion and metastasis is clearly warranted.

### Epithelial-mesenchymal transition in promoting PCa metastasis

The steps that lead to PCa metastasis (Figure 1) include degradation of the ECM, detachment of the cancer cells from the ECM as well as their detachment from each other, migration toward and subsequent entry into the blood or lymphatic system (89–91). The machinery and signaling pathways used in these invasive events are part of the normal wound healing response of epithelial tissues. Upon tissue damage, a host of growth factors and cytokines are released from the blood stream that activate the stromal fibroblasts (PDGF, TGF-β), endothelial cells (VEGF, FGF, IL8), and epithelial cells (HGF, TGF-β) to repair the tissue and fill in the wound (92). Through these signals epithelial cells are forced to loosen their matrix adhesions via activation of proteases and integrin signaling, and are induced to migrate across matrix being remodeled by the stroma. Some may even detach from each other to facilitate filling in the wound. It is this latter event, loss of cell–cell adhesion and depolarization of epithelial cells that is thought to trigger the final conversion of primary cancer cells into metastatic ones. Once loosened from the matrix and from each other, these cells are now free to roam if they have acquired the proper mutations that allow them to survive as non-adherent cells.

This process is referred to as epithelial–mesenchymal transition (EMT), where the loosened epithelial cells take on the physical properties of mesenchymal fibroblast-like cells (92). The classical marker of EMT is cadherin switching; where E-Cadherin expression is lost and N-Cadherin is gained (89, 90). E-cadherin, which promotes homotypic binding between two adjacent epithelial cells, prevents the cancer cells from breaking away from each other and reinforces tight junctions to preserve epithelial barrier function and apical/basal polarity (90, 93). The metastasis suppressor, tetraspanin CD82, promotes E-cadherin-based cell–cell adhesion, and suppresses integrin-based migration (51). Loss of this crucial regulator reduces cell–cell adhesion, while at the same time promoting enhanced migration; thus, its loss would strongly facilitate an EMT-like phenotype. Decreased CD82, E-Cadherin, or β-catenin (the anchoring protein for E-cadherin) is associated with poor PCa prognosis (23, 46, 94, 95). TGF-β is the most classical inducer of EMT, signaling through Smad family transcription factors to induce the expression of the EMT-regulating transcription factors Snail, Slug, Zeb-1, and/or Twist (96–100). These EMT-associated transcription factors, through interactions with epigenetic regulators, control expression of genes involved in cell polarity, cell–cell contact, cytoskeleton structure, and ECM degradation (Figure 1), including repression of the E-cadherin gene (101).

While EMT is well-established in several other epithelial cancer types, its specific role in PCa remains controversial. This is complicated by the histological data demonstrating that PCa remains very epithelial-like in both primary tumors and in metastatic tissues; expressing E-cadherin and other classical prostate epithelial markers (94, 102). Yet, a few isolated tumor cell lines, especially those that have lost AR expression, readily adapt EMT phenotypes and are relatively invasive and metastatic (102–104). The necessary presence of AR in PCa tumors, which drives a luminal differentiation phenotype may make the conversion to EMT a relatively difficult process (103). It might explain the long latency for conversion of primary PCa to metastatic disease (105). The heterogeneous display of EMT phenotypes in PCa and other human tumor samples, has fueled the hypothesis that EMT is a transient process necessary for escape and dissemination, that reverses back, i.e., MET (mesenchymal epithelial transition) when cells reach their distant sites. A study that tested this hypothesis using a Tet-inducible model, demonstrated that transient induction of EMT was required for the migration of squamous carcinoma cells out of the skin into the blood stream, and subsequent shut off was required to establish metastatic tumors in the lung (106). If this happens in PCa is not clear, and the factors that drive it are even less clear.

The soluble factors that are secreted into a wound by physical disruption are the same factors present in the tumor microenvironment. Transforming growth factor-β (TGF-β) is a potent inducer of EMT and is released by many cellular components of the tumor microenvironment. However, the ability of primary tumors to respond to TGF-β is hindered by the normal growth inhibitory effects of TGF-β signaling, and thus the tumor cells typically block this pathway. So the cells must find ways to reactive some aspect of TGF-β signaling that doesn’t cause growth suppression or find other indirect ways to stimulate EMT. One study suggested that direct cell–cell contact between tumor cells and platelets synergistically cooperated with TGF-β, to directly activate NF-κB signaling in the tumor cell to promote EMT and metastasis (107).

Inflammation induced during wound healing and in tumors also impacts the behavior of tumor cells. Many studies have demonstrated that wounding is a tumor-promoting event, especially chronic wounding where inflammation is high (92). The role of inflammation in PCa initiation was originally identified histologically by the presence of prostate inflammatory atrophy (PIA) in tumor samples (108). Recent mouse studies demonstrate that prostatic inflammation induced by prostitis, enhances basal-to-luminal differentiation and accelerates the initiation of PCa (109). Two sources for inflammatory signaling in PCa have been proposed, the stromal cancer-associated fibroblasts (110) or mesenchymal stem cells (111). A recent study highlighted the importance of adipocytes in inducing inflammatory responses in PCa cells within the bone through the lipid chaperone FABP4, triggering IL-1β expressing and oxidative stress protein HMOX-1 (112). Other studies proposed the inflammatory responses in PCa are mediated through NF-κB signaling (113). Inflammatory cytokines like TNFα and interleukins produced by both tumor cells and surrounding cells (114) activate NF-κB signaling and one consequence of this is the release of TGF-β (115, 116).

Non-TGF-β pathways can also activate EMT. TNFα can act independently of TGF-β to induce EMT by repressing GSK-3β, activating the AKT pathway, and stabilizing Snail (117–119). Wnt signaling also stimulates EMT in PCa cells. Expression of SOX2 induces EMT, and this was shown to be mediated by SOX2 binding to and activating β-catenin (120). The PCa-specific fusion and oncogene, TMPRSS2-Erg, enhances cell invasion (121, 122). Manipulation of the fusion gene in VCaP cells, altered Frizzled4 (Fzd4) expression (123). Fzd4 signaling promoted cell adhesion-related EMT phenotypes in VCaP cells. HIF1α, activated under hypoxic conditions, promotes aggressive tumor cell invasion and metastasis (124, 125). Overexpression of HIF1α in some PCa tumor cell lines promoted EMT that was dependent on β-catenin (126).

Further demonstration that EMT/MET phenotypic conversions are essential for the progression and metastasis of PCa to the bone is highly warranted. While current mouse models have been effective at defining specific molecular events occurring within the primary tumor or bone-resident tumors, the process whereby a confined prostate tumor is converted to a metastatic bone tumor has not been adequately modeled. The models do not currently reflect what is observed in human patients; i.e., AR-positive epithelial cells that migrate to and take up residence in the bone to induce an osteoblastic bone reaction. Understanding the specific genetic and epigenetic alterations that promote EMT-like phenotypes in PCa will be important to understanding the switch between indolent and lethal PCa, improving staging and prognosis of PCa patients and preventing over treatment.

## Tumor Microenvironment in Prostate Cancer Bone Homing and Colonization

### Secreted factors in PCa bone homing

**Figure 2:**
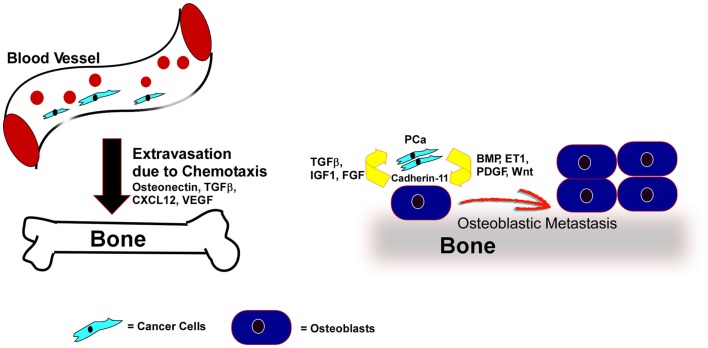
**PCa cells home to bone by chemo-attractants and colonize through direct association with osteoblasts, where the PCa cells secrete factors that promote osteoblastic responses and the osteoblasts reinforce tumor cell survival and growth**. Expression of various chemo-attractants (Osteonectin, TGFβ, CXCL12, VEGF) guide PCa cells to extravasate and home toward the bone. Once in the bone-microenvironment the cancer cells interact with bone-forming osteoblasts via Cadherin-11. Factors like BMP, ET-1, Wnt, or PDGF, secreted from the cancer cells promote the proliferation and differentiation of osteoblasts. In turn the bone-microenvironment secretes soluble factors like FGF, IGF, and TGFβ to promote tumor cell survival and proliferation.

Different types of cancers develop metastases in very specific organs. Several secreted factors have been proposed to promote organ-specific homing (Figure 2). A few of these chemotactic signals can attract cancer cells toward the bone. Conditioned media from osteoblasts differentiated *in vitro* served to induce migration and invasion of breast and melanoma cells, indicating osteoblasts secrete potent factors that can induce metastasis to the bone (127, 128). Osteonectin, a purified active factor from the bone, promoted invasion of bone-metastatic cancer cells, but not the non-bone-metastatic cancer cells indicating that bone possess chemotactic factors that can promote tissue-specific homing of cancer cells (129). It was demonstrated that osteoblast conditioned media containing higher amounts of TGF-β promoted chemotaxis and invasion of PC3 cells. Given the abundance of TGF-β in the bone environment, it could act as a chemo-attractant of PCa cells to the bone (130).

Chemokines and cytokines have chemo-attractant properties that play an important role in the cancer cell proliferation, survival, and gene transcription. Chemokine receptors are involved in many processes of cancer metastasis (131, 132). Mice bearing autoimmune arthritis have higher incidence of breast cancer metastasis to the bone, which was proposed to be due to the presence of higher amounts of circulating levels of pro-inflammatory cytokines in these autoimmune arthritic mice (133).

The chemokine receptor, CXCR4, and its ligand CXCL12/SDF are widely studied in PCa bone metastasis (134, 135). In bone marrow, CXCL12 is expressed in osteoblasts, fibroblasts, and endothelial cells (136). Blocking CXCR4 in PCa cells using neutralizing antibody inhibited the dissemination and colonization of PCa cells in mice tibia following intra-cardiac injection (135). Akt1 reportedly induces the expression of CXCR4 in PTEN-null PCa cells, and overexpression of Akt-1 promoted intra-tibial tumor growth of PCa cells (137). These results indicate that Akt-1 might be inducing the CXCR4/CXCL12 axis and thus promoting PCa metastasis. PCa cells home toward areas in the bone marrow rich in osteoblasts where the hematopoietic stem cell (HSC) niche resides. In fact, PCa cells can bind to and displace mouse HSCs from the niche. Furthermore, the cancer cells egress out of the HSC niche into the blood when CXCR4 signaling is blocked by AMD3100 (138). These findings suggested that the CXCL12/CXCR4 axis is important for chemotaxis of PCa cells to the bone. However, inhibiting CXCR4 with CTCE-9908, a drug approved by FDA for osteosarcoma, inhibited spleen, liver, and lymph node metastasis of PCa cells, indicating CXCR4 may be a common metastatic factor, rather than one that is bone specific.

### Ectopic site pre-remodeling

The famous “Seed and Soil” hypothesis, put forward more than 100 years ago by Dr. Stephen Paget, was used to explain why different types of cancer preferentially metastasize to different specific tissues. The theory proposed that distant organs, like the bone, provide a preferred “fertile soil” for cancer cells, and the cancer cells were preferentially attracted to that tissue. However, Isaiah Fidler’s group demonstrated that tumor cells were present in vasculature of all the organs, yet metastasis only developed in certain organs but not in others (139, 140). David Lyden’s group put forward the pre-metastatic niche model, where remodeling of only the preferred ectopic site(s) for metastasis occurs much earlier, before the cancer cells even break away from the primary tumor (139).

The niche remodeling events, mediated by soluble factors acting on non-cancer cells, govern the route of dissemination of cancer cells to a specific microenvironment. They demonstrated that bone marrow-derived hematopoietic progenitor cells expressing VEGF receptor 1 (VEGFR-1), homed to the specific metastatic sites through integrin α4β1. At the same time, the tumor cells secrete factors that induce the fibroblasts within the pre-metastatic niche to secrete Fibronectin, an α4β1 ECM ligand. The VEGFR1-positive cells then promote chemo-attraction and adherence of circulating tumor cells (141). Consistent with this idea, Hirutsuka et al. (142) demonstrated in a mouse model of melanoma metastasis that VEGF-A, TGF-β, and TNF-α released from the primary tumors induced the expression of chemokines in the lung parenchyma but not in other organs. In another study, persistent STAT3 activation was detected in distant organs such as the lung before tumor cell arrival. S1PR1–STAT3 up-regulation in tumor cells induced S1PR1–STAT3 at these distant sites and in myeloid cells. Ablation of STAT3 in the myeloid compartment inhibited STAT3 activity in the lungs, inhibited formation of pre-metastatic niche, and inhibited lung metastasis (143). Whether a similar pre-metastatic niche remodeling and non-tumor cell signaling governs PCa bone metastasis is not known. However, a past study demonstrated enhanced bone metastasis of orthotopic xenografted human cell lines upon androgen ablation (144). ADT in human PCa patients induces bone loss that is often corrected by bisphosphonates (145). Similarly, castrated mice lose bone mass (146). The full contribution of ADT to pre-metastatic niche conditioning, bone metastasis, osteoblastic reactions, or emergence of castration-resistant disease, as a result of bone-microenvironment interactions needs further investigation.

### Adhesion molecules in PCa colonization

Several studies have suggested that the choice of metastatic organ is not necessarily dictated at the time tumor cells escape the tumor and end up in the circulation. Circulating tumor cells can be found in patients who do not have or do not develop metastatic disease (147). Furthermore, tumor cell entry is not restricted to specific organs, majority of circulating tumor cells extrude into tissues (147, 148). A PCa study demonstrated that 50% of the patients with primary tumors had circulating tumor cells as well as tumor cells lodged in the bone (149), yet only 12–13% of these patients ever develop metastatic disease. Thus, additional events are required for the tumor cells to grow and colonize the metastatic site.

Cadherin-11, commonly known as osteoblast cadherin, is primarily found in osteoblasts, with very low but detectable expression in brain, testis, and lung (150, 151). It is an adhesion molecule that mediates many steps of osteoblast maturation (150, 151). Cadherin-11 expression is increased in metastatic PCa compared to primary tumors but is not present in normal prostate tissue. Furthermore, elevated Cadherin-11 was found in human PCa bone metastasis relative to lymph node metastasis (152), indicating cadherin-11 is specifically associated with bone metastasis. Cadherin-11 may mediate the binding of cancer cells to osteoblasts (Figure 2). Such binding might also promote cross talk between cancer cells and osteoblasts and induce osteoblastic lesions. Intra-cardiac injection of PC3 cells expressing Cadherin-11-specific shRNA displayed a significant decrease in bone metastasis compared to the control cells (152). PCa cells derived from bone express high levels of cadherin-11, and expression of cadherin-11 in PCa cells promoted PCa cell invasion and migration and increased the adhesion and intercalation between osteoblasts in an *in vitro* culture model (153). Another group demonstrated that bone-tropic MDA-MB-231 breast cancer cells also express high levels of cadherin-11 compared to brain-tropic MDA-MB-231 cells. Thus, cadherin-11 is likely an important determinant of bone-tropism in cancer cells (154).

## Tumor Microenvironment in Promoting Osteoblastic Lesions

Breast cancer metastasis is usually osteolytic (bone degrading); however, PCa is osteoblastic, i.e., leading to new bone formation (155, 156). It has been reported that some colon and cervical cancers are also osteoblastic (157). The exact mechanisms by which osteoblastic versus osteolytic metastases occur is still unclear. However, the differences are likely to reside in the differential interaction of tumor cells with the bone microenvironment. Bone, a dynamic connective tissue is constantly remodeling during an individual’s lifetime. The process of remodeling is dependent on two cells types, osteoblasts and osteoclasts, both of which work in harmony to maintain the normal bone. Osteoblasts derived from mesenchymal stem cells in the bone marrow, make new bone. Whereas osteoclasts, which are modified macropages derived from monocytes, degrade bone (84). During osteoblastic metastasis, the bone remodeling favors bone formation over resorption.

### Factors that induce osteoblastic bone metastasis

The number of osteoblasts surrounding PCa cells is increased in osteoblastic bone metastasis (155). However, the newly formed bone is weak and fragile, lacking mechanical strength. These bones are composed of randomly orientated and loosely packed collagen bundles, resulting in weak bone strength and frequent fracture (158). Furthermore, the excessive bone growth disrupts the bone marrow compartment, reducing immune function. Many factors can induce the growth and differentiation of osteoblasts and their precursors (Figure 2). Tumor cells secrete many of these factors, and thus may actively promote osteoblastogenesis.

#### Bone morphogenetic proteins

Bone morphogenetic proteins are members of the TGF-β superfamily and various isoforms of BMP promote both prostate and breast cancer metastasis (159, 160). One major source of BMP expression appears to be from the tumor cells. Elevated expression of both mRNA and BMP-6 protein is detected in primary PCa tissues and in PCa cell lines (161, 162). BMPs could influence metastasis by acting directly on the tumor cells or through their effects on the bone microenvironment. For instance, BMP-2 induces resistance to apoptosis due to hypoxia (163) and promotes breast cancer cell invasion and migration (164); whereas, BMP-6 promotes migration and invasion of PCa cells (161). BMP-2 and -7 stimulated cellular migration and invasion of PCa cells (165), and BMP-6 acting through Smad signaling directly induced the transcription of extracellular proteases such as MMP-1 and 9, required for invasion (159).

Findings from genetically modified mice demonstrate that the normal role of BMPs in the bone is to induce the differentiation of osteoblasts (166, 167). For example, BMP-7 knockout mice have smaller skeletons and reduced mineralized bone (168). The ability of BMP-7 to control bone mineralization or osteoblast differentiation can be attributed to the induced expression of crucial differentiation factors, Runx2 and Osterix, in bone stromal cell precursors (169, 170). High BMP-7 expression was detected in PCa-induced bone lesions, while its expression in primary tumors is low (155, 171). However, its exact role in promoting osteoblastic vs. osteolytic lesions remains controversial. Nonetheless, BMP-7 produced by tumor cells has the potential to impact osteoblast differentiation within PCa bone lesions. The most intriguing aspect of BMP-7 is that while its expression is controlled by androgen and it is required for normal prostate development, the most elevated BMP-7 expression was observed in castration-resistant tumors within the bone (171). However, BMP-7 expression in prostate bone tumors appears to be largely growth suppressive and may promote PCa cell dormancy (172).

Elevated BMP-6 expression is also associated with PCa bone metastasis (159). BMP-6 produced by cancer cells was able to induce mineralization of M3T3 pre-osteoblasts, and blocking BMP-6 activity reduced osteolastic lesion formation by LuCaP 23.1 cells *in vivo* (161). Two studies demonstrated that Wnt5a or Wnt3a, generated by bone stroma cells, induces the expression of BMP-6 in PCa cells (173, 174). This was mediated by non-canonical JNK and canonical β-catenin signaling pathways, respectively (174). The induction of BMP-6 by Wnt5A secretion occurred in the absence of androgen and promoted androgen-independent growth of the tumor cells (173). Interestingly, in a recent patient-derived bone xenograft model, transplantation of human bone-metastatic tumors into the bone, but not in the skin, resulted in castration-resistant tumor growth (175). Thus, the bone microenvironment has a high capacity to influence PCa treatment. The BMPs secreted by PCa cells through Wnt signaling, in turn induce the differentiation of osteoblasts. For instance, BMP-4 produced by PCa cells promoted osteoblast differentiation of mouse stromal cells, as measured by the production of alkaline phosphatase, osteocalcin, and collagen type II. At the same time, BMP-4 also stimulated the production of sonic hedgehog (Shh) by the tumor cells (176). Shh stimulated Smad1 and BMP receptor expression in the mouse stromal cells, which enhanced their response to BMP-4. The ability of Shh to induce osteoblast differentiation was promoted by collagen production and was Gil1-dependent, but Runx2-independent (177, 178). Thus, BMPs and Shh cooperatively provided cues for the growth of PCa cells and the differentiation of bone stromal cells. All these results indicate that BMPs and Shh from the PCa cells play an important role in inducing osteoblast differentiation from bone stromal cells, and thus likely contribute to osteoblastic bone phenotypes.

#### Endothelin 1

Endothelin 1, a potent vasoconstrictor along with other family members ET-2 and ET-3, is produced by the vascular endothelium. Plasma levels of ET-1 are elevated in several types of cancers, including PCa (179). ET-1 inhibits PCa cell apoptosis via enhanced Bcl-2 family member expression and PI3K/Akt activation (180). In ovarian carcinoma cell lines, ET-1 promotes invasion via the activation of extracellular proteases like MMP-1 (181). In addition to promoting cancer metastasis, ET-1 promotes osteogenic properties; ET-1 null mice exhibit hypoplasia of facial bones (182). ET-1 is a potent mitogenic factor of osteoblasts and patients with osteoblastic bone lesions have increased serum levels of ET-1 (171, 183). Elevated ET-1 might contribute to osteoblast proliferation and differentiation (184–186).

In clinical trials, atrasentan (endothelin A receptor antagonist) suppressed bone remodeling in castration-resistant metastatic patients (187). However, atrasentan in combination with docetaxel, a chemotherapeutic agent, did not improve progression-free survival in castration-resistant bone-metastatic patients (188). One of the potent pathways by which ET-1 promotes osteoblast activity is through the activation of Wnt signaling via the inhibition of the Wnt suppressor DKK1 (189). ET-1 inhibited DKK-1 expression, but also increased the expression of Type 1 collagen, a predominant protein constituent of bone matrix (185, 189).

#### Wnt

Wnts constitute a family of 19 secreted glycoproteins, whose dysregulation plays an important role in the progression of many cancers including breast, gastric, prostate, melanoma, and glioblastoma (190, 191). Wnt ligands bind to a seven-pass transmembrane receptor composed of Lrp5/6 and frizzled genes to transduce signals to the cytoplasmic protein Disheveled (Dsh). Dsh blocks GSK-β to inhibit β-catenin phosphorylation, by disrupting the β-catenin/Axin complex, leading to β-catenin stabilization, and its nuclear translocation to interact with TCF/LEF transcription factors (192, 193). Loss of APC activates the Wnt pathway by stabilizing β-catenin. Loss-of-function of APC mutations is common in many cancers (193–195). Increased nuclear β-catenin expression correlated with advanced, metastatic, and hormone-refractory prostate carcinoma (196). Upregulation of the Wnt pathway by means of increased Wnt secretion, decreased expression of inhibitors such as APC, sFRP, DKK1, or Wif1, or constitutive activation of β-catenin, induces the activation of downstream target genes like c-Myc, c-Jun, and various other genes important in both cancer development and metastasis (91, 192, 193). MMP-14, which promotes invasion and metastasis of cancer cells, is also a direct target of β-catenin/TCF signaling (197). The exact mechanisms that lead to elevated β-catenin in PCa are not clear, but it is not usually due to APC mutation (194).

Within the bone, Wnt signaling promotes osteoblast differentiation by directly stimulating Runx2 expression in osteoblasts both *in vivo* and *in vitro* (198). Wnt signaling also stimulates BMP-2 expression, inducing the trans-differentiation of non-osteogenic cells into osteoblasts (199). Wnt signaling may contribute to the osteoblastic phenotype. Blocking DDK1 expression in PC3 cells, which releases the block on Wnt signaling, switched the normal osteolytic phenotype induced by PC3 cell to osteoblastic. Conversely, overexpressing DKK-1 in C4-2B cells converted the normal mixed lesion to an osteolytic lesion (200). Thus, Wnt signaling contributes to PCa osteoblastic bone lesions. Further investigations in the specific components of the Wnt pathway involved, and determining if they also contribute to metastasis *per se* will be important.

#### Platelet-derived growth factor

Platelet-derived growth factor, a potent growth factor which plays an important role in tumor progression, consists of disulfide-bonded homodimer polypeptide chains of A, B, C, D, and heterodimer AB (201). Aberrant signaling through PDGF receptors promotes progression of many tumors, including PCa. PDGFRβ, which is frequently activated in bone-metastatic PCa patients, is activated both by PDGF-B and PDGF-D (202). PDGF-D, which promotes PCa cell proliferation and tumor growth, is overexpressed in prostate tumors with increasing Gleason score (203). Furthermore, PDGF also stimulates the interaction of PCa cells with bone stromal cells. In the bone microenvironment, PDGF is synthesized by platelets, macrophages, osteoclasts, endothelial cells, and all cells differentiating from mesenchymal stem cells, including pericytes, and osteoblasts (204). Thus, PDGF has the potential to act as a central connector for many interactions within the bone microenvironment that influence tumor growth. Blocking PDGF receptor signaling inhibits the growth of human breast and pancreatic cancer in bones (205, 206), and subsequently reduces bone resorption. However, a phase I clinical trial with a potent PDGFR inhibitor, imatinib mesylate in combination with docetaxel, in castration-resistant PCa patients with bone metastasis did not show any improvement in the median progression-free survival of patients, as compared to docetaxel alone (207). However, imatinib mesylate inhibits many other kinases, like Abl and c-Kit (208). Whether PDGFR inhibitors in combination with the second generation anti-androgens, Enzalutamide or abiraterone, will be more effective should be investigated.

### Factors released from the bone microenvironment

#### Transforming growth factor-β

Transforming growth factor-β is one of the most abundant cytokines which induces osteoblast proliferation but inhibits its differentiation (209, 210). The effects of TGF-β on osteoclasts are controversial. Most of the studies investigating TGF-β in cancer bone metastasis have focused on osteolytic bone metastasis of breast and cancers other than PCa. A “vicious cycle” model has been proposed to explain TGF-β signaling and osteolytic bone metastasis. TGF-β, signaling through Gil2, in a Hedge Hog-independent manner, stimulates the expression of parathyroid hormone-related protein (PTHrP), which activates osteoclasts (211). In turn, more TGF-β is released after bone resorption, which further enhances cancer growth and osteoclast activation, initiating a “vicious cycle” (212, 213). Inhibition of TGF-β decreases osteolytic lesions induced by breast and melanoma cancer cells in mouse tibia (214–216). In the first report on the role of TGF-β in PCa bone metastasis, it was found that loss of TGF-β responsiveness in the fibroblasts induced the up regulation of CXCL16 and CXCL1, which promoted PCa cells adhesion to the bone matrix, and promoted mixed (osteoblastic/osteolytic) metastatic lesions (217). Thus, it will be important to further investigate the cell-specific role of TGF-β in the bone microenvironment on PCa osteoblastic bone metastasis.

#### Insulin-like growth factor

High serum levels of insulin-like growth factor are associated with higher risk of breast, prostate, and colorectal cancer. Signaling through IGF1R promotes cell proliferation, apoptosis, and invasion of cancer cells; which are all integral steps in cancer metastasis (218). For example, inhibition of IGF-1R diminishes the invasion of PCa cells and also inhibits expression of MMP-2, an extracellular protease necessary for invasion (219). The ability of IGF-1 to induce PCa cell proliferation and survival is dependent on loss of Pten, a tumor suppressor commonly lost in PCa (220). However, IGF is another important coupling factor in the bone, activating both bone formation and resorption. IGF, which promotes proliferation, invasion, and metastasis of cancer cells is released during bone resorption (84). One interesting study showed that neutralizing antibody to IGF, but not antibody to TGF-β, FGF, or PDGF, blocked the breast cancer anchorage-independent growth induced by resorbed bone extract; further supporting a unique role for IGF-1 in bridging the cross talk between the bone microenvironment and the cancer cells (221). Bone-derived IGF promoted bone metastasis of breast cancer cells by stimulating proliferation and inhibiting apoptosis of cancer cells (221). Elevated IGF-1 receptor expression in the stroma surrounding clinical PCa samples correlates with high Gleason score (222). Thus, IGF-1 released from the bone could stimulate the stroma to support PCa growth. Indeed, blocking IGF-1 and IGF-1 receptor inhibits PCa growth in the bone and reduced the osteoblastic bone formation. Thus, IGF-1 signal inhibition could be strategy for limiting PCa bone metastasis.

#### Fibroblast growth factor

Fibroblast growth factor, a family of ubiquitously expressed and secreted factors, regulates processes like development, wound healing, and neoplastic transformation through mitogenesis and angiogenesis. Increased expression of some growth factors from the FGF family and their receptors are reportedly associated with PCa progression. Aberrant activation of FGF receptors (FGFR) induces the activation of downstream targets like PI3K, MAPK, and STAT3, all of which play an important role in the progression of PCa (223–225). One of the FGF family members, FGF-1 (acidic FGF) is expressed in a majority of PCa and its expression is associated with high Gleason score (226). Levels of FGF-2 (basic FGF) and FGF-6 are also elevated in PCa tissues as compared to normal (227, 228). In one transgenic mouse model, FGFR1 overexpression led to PIN (229), but in another model, activated FGFR1 promoted adenocarcinoma and metastasis to lymph nodes and liver (230). FGF may promote PCa metastasis via regulation of cell survival (231). PCa bone metastases express FGF-8 and/or FGF-9, and both of these FGFs are reported to promote osteoblast differentiation and new bone formation (232–234). FGF-9 expression is also associated with high Gleason score and neutralizing antibody against FGF-9 inhibited bone formation and bone lesions in mice (232, 235). On the other hand, other family members, like FGF-7 and FGF-10, are required for normal differentiation of normal prostate epithelial cells (236). Nonetheless, overexpression of FGF-7 in a transgenic mouse prostate epithelium led to PIN (237), and overexpression of FGF-10 in the mesenchyme in the prostate regeneration mouse model was sufficient to induce multifocal PIN had low-grade PCa (238). Thus, the context and level of FGF signaling may differentially impact PCa development and progression. The mechanisms that underlie the differential expression of FGF members and their respective receptors to promote or inhibit PCa growth in different microenvironments, and the cellular constituents upon which it acts need to be better resolved in PCa.

## Challenges That Remain

The high dependence of host environmental factors on metastatic processes necessitates the use of animal models to clarify and demonstrate that dependency. The most commonly used model is the mouse, which has a physiology and prostate organ structure that is significantly different from human. Nonetheless, valuable insight can be gleaned from the proper models. Unfortunately, for PCa researchers many of the current mouse models fail to fully recapitulate human disease progression; i.e., an AR-dependent tumor growing in the prostate gland that spontaneously metastasizes to the bone to from an AR-dependent osteoblastic cancer.

Models using genetic introduction of known PCa-associated mutations into the mouse genome, rarely metastasize to the bone. The few models that do are neuroendocrine or use mutations not reported to be present in human disease. Nonetheless, one unifying theme in the models that do produce significant metastases is the abrogation of p53. While p53 loss is reported in <20% of metastatic PCa tumors, other mutations that indirectly alter p53 function remain a strong possibility. Another tumor suppressor, Pten, whose homozygous loss strongly correlates with metastatic progression in human cancer, but whose loss alone in the mouse does not lead to metastatic bone disease, may also contribute to metastasis. Interestingly, both p53 and Pten impact chromosomal stability (239), disruption of which is thought to be necessary for the selection of genetic variants within the tumor that eventually evolve the capacity to metastasize. Recent studies suggest that EMT-associated transcription factors suppress p53 signaling and impact DNA repair; thus, linking disease progression by the tumor microenvironment with chromosomal instability (106). Identifying the factors that specifically lead to chromosomal instability, the genetic alterations within PCa that promote metastasis, and how the tumor microenvironment influences this, has the potential to significantly increase our understanding of metastatic conversion in PCa.

Several metastatic PCa cell lines isolated from bone metastases, either from human samples or mouse xenografts (VCaP, C4–2, PC3), retain high capacity to grow when implanted directly into the bone, or in a few cases when injected into the heart. Two PCa lines, one isolated from the brain, DU145, and one from ascites, ARCaP, can also grow when implanted in bone; however, the LNCaP line isolated from a lymph node grows poorly in the bone. Most of them, when implanted orthotopically in the prostate, do not metastasize to the bone; though some make it to the lung. ARCaP was reported to metastasize to the bone after orthotopic injection (240). This was accompanied by EMT conversion. Only a few of these, when implanted in the bone, make osteoblastic lesions (DU145, C4–2, ARCaP). Thus, whatever properties these cells once had in the human host that permitted their full metastatic progression in humans has been lost. Furthermore, at least 3 of these lines, PC3, DU145, and ARCaP express AR at such low levels they do not use the AR-regulated pathways seen in over 90% of human PCa metastases. The LuCaP series of human xenografted and SubQ-passaged tumors (241), isolated primarily from soft tissue metastases and still expressing AR, can grow and form osteoblastic lesions when implanted in the bone. Removal of primary LuCaP tumors following orthotopic injection, allowed the development of micrometastases to lymph nodes and soft tissues (242), but not bone. Thus, these studies indicate that many PCa tumor cell lines, whether they originally came from bone or not, have a high capacity to grow in bone. However, they all lack the ability to home to bone from the prostate, and their capacity to induce osteoblastic lesions is variable. Developing human lines or mouse models that can display the full metastatic progression, either through selection or genetic manipulation, remains the Holy Grail for understanding PCa metastasis and having models that can be used for effective therapy development and testing.

## Conclusion and Perspectives

Metastasis is the major cause of PCa death. Understanding how cancer cells metastasize toward the bone is needed to design drugs that prevent or interfere with PCa metastasis. Many studies suggest that EMT transcription factors drive the initial phases of PCa metastasis, although the events, either within the tumor or contributed by the tumor microenvironment, that trigger EMT specifically in PCa are still not known. Overexpression of EMT transcription factors, in conjunction with responses to host-derived chemotactic factors, might lead to PCa-specific homing and metastasis to the bone. Once PCa is disseminated in the bone, the cross talk between the bone microenvironmental factors and the PCa tumor cells contribute to the establishment of osteoblastic lesions. The factors mediating this cross talk and their signaling pathways need to be further delineated to ultimately halt the progression of metastatic lesions in the bone. The development of better animal models that fully recapitulate the metastatic process as seen in human disease is paramount to deciphering the molecular events associated with PCa metastasis.

## Author Contributions

Dr. Sourik S. Ganguly drafted, helped revise, and designed the figures for the manuscript. Dr. Xiaohong Li revised and provided intellectual content. Dr. Cindy K. Miranti revised, provided intellectual content, and finalized the manuscript.

## Conflict of Interest Statement

The authors declare that the writing of this review article was conducted in the absence of any commercial or financial relationships that could be construed as a potential conflict of interest.
